# The importance of comparative phylogeography in diagnosing introduced species: a lesson from the seal salamander, *Desmognathus monticola*

**DOI:** 10.1186/1472-6785-7-7

**Published:** 2007-09-07

**Authors:** Ronald M Bonett, Kenneth H Kozak, David R Vieites, Alison Bare, Jessica A Wooten, Stanley E Trauth

**Affiliations:** 1 Museum of Vertebrate Zoology and Department of Integrative Biology, University of California at Berkeley, Berkeley, CA, 94720, USA; 2 Department of Ecology and Evolution, Stony Brook University, Stony Brook, NY, 11794, USA; 3 Department of Biological Sciences, Arkansas State University, State University, AR, 72467, USA; 4 Department of Biological Sciences, University of Alabama, Tuscaloosa, AL, 35487, USA

## Abstract

**Background:**

In most regions of the world human influences on the distribution of flora and fauna predate complete biotic surveys. In some cases this challenges our ability to discriminate native from introduced species. This distinction is particularly critical for isolated populations, because relicts of native species may need to be conserved, whereas introduced species may require immediate eradication. Recently an isolated population of seal salamanders, *Desmognathus monticola*, was discovered on the Ozark Plateau, ~700 km west of its broad continuous distribution in the Appalachian Mountains of eastern North America. Using Nested Clade Analysis (NCA) we test whether the Ozark isolate results from population fragmentation (a natural relict) or long distance dispersal (a human-mediated introduction).

**Results:**

Despite its broad distribution in the Appalachian Mountains, the primary haplotype diversity of *D. monticola *is restricted to less than 2.5% of the distribution in the extreme southern Appalachians, where genetic diversity is high for other co-distributed species. By intensively sampling this genetically diverse region we located haplotypes identical to the Ozark isolate. Nested Clade Analysis supports the hypothesis that the Ozark population was introduced, but it was necessary to include haplotypes that are less than or equal to 0.733% divergent from the Ozark population in order to arrive at this conclusion. These critical haplotypes only occur in < 1.2% of the native distribution and NCA excluding them suggest that the Ozark population is a natural relict.

**Conclusion:**

Our analyses suggest that the isolated population of *D. monticola *from the Ozarks is not native to the region and may need to be extirpated rather than conserved, particularly because of its potential negative impacts on endemic Ozark stream salamander communities. Diagnosing a species as introduced may require locating nearly identical haplotypes in the known native distribution, which may be a major undertaking. Our study demonstrates the importance of considering comparative phylogeographic information for locating critical haplotypes when distinguishing native from introduced species.

## Background

Species introduced by human activities are one of the leading threats to biodiversity [1-4]. A critical step in ameliorating the impacts and spread of introduced species is to identify and contain them in their infancy [5,6]. However, humans have been altering biotic patterns across the world since the "Age of Exploration" [7], and for many regions species introductions precede complete biodiversity inventories, obscuring our ability to distinguish introduced from native flora and fauna. This is potentially a very important yet time sensitive distinction, especially for isolated populations, because the alternate diagnoses suggest opposite conservation action. Isolated native populations may need special protection [8-10], while introduced species may need to be eliminated [5,6]. In the absence of historical information genetic data may be necessary to determine the origin of an isolated population, although pin-pointing the closest relatives in the known native distribution (to test the history of the isolate adequately) can be an endless task. Here, we show that using comparative phylogeographic patterns of taxa that are co-distributed across the known, native range of a putatively introduced population may be the most effective strategy for distinguishing native from introduced species.

**Figure 1:**
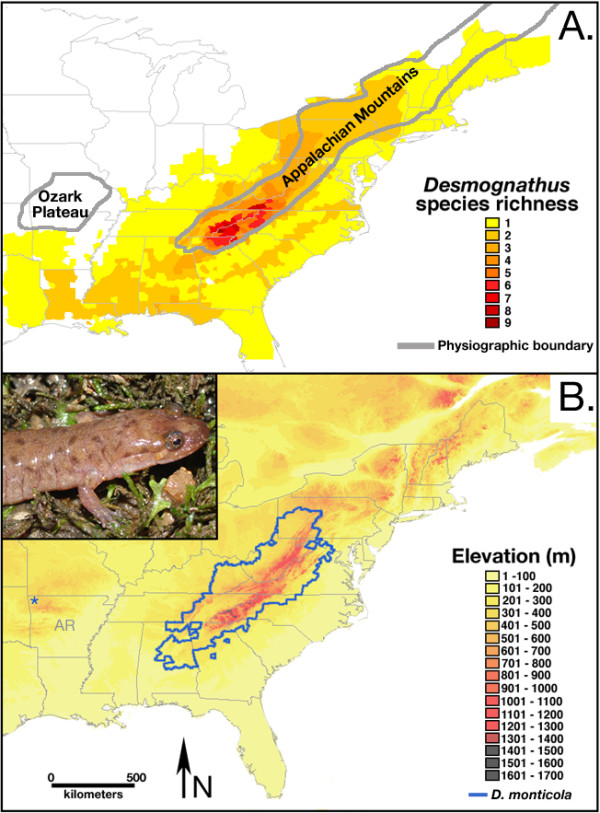
**Distribution and species richness of *Desmognathus***. **A. **Species richness of the genus *Desmognathus*, with the highest species diversity in the southern Appalachian Mountains. Note: *Desmognathus *are absent from the Ozark Plateau. **B. **Geographic distribution of *Desmognathus monticola *outlined in blue overlaid on an elevation map of eastern North America. The isolated population of *D. monticola *in the Ozarks is designated with a blue star. Inset is a photograph of an adult *D. monticola *from the Ozarks.

The Plethodontidae is the most species rich family of salamanders [11,12], and their diversity has been primarily shaped by allopatric speciation events resulting in an abundance of cryptic species and genetically distinct isolated populations [e.g., [13-16]. The plethodontid genus *Desmognathus*, endemic to eastern North America, includes 19 recognized species; their primary diversity is centered in the southern portion of the Appalachian Mountains [11,12] (Figure 1A). This genus is marked by great ecomorphological diversity, ranging from small, strictly terrestrial species to very large-bodied, stream-dwelling predators [17-19], but most ecomorphs also contain parapatrically distributed cryptic species [20-22].

The Ozark Plateau of east-central North America is a karst uplift separated from the Appalachian Mountains by the low elevation flood plain of the Mississippi River. The Ozark Plateau harbors many endemic species, including a unique salamander fauna [23,24]. There are only a few historical reports of *Desmognathus *on the Ozark Plateau [25,26], but no credence has been given to these records [27,28] since no museum vouchers exist and no subsequent specimens were found for the next 40+ years. In 2003, a very restricted, but thriving, population of *Desmognathus *was discovered in the western Ozarks [29]. This population is similar, both morphologically and mitochondrially, to the seal salamander (*Desmognathus monticola*), a large stream-dwelling species that is widespread in the Appalachian Mountains, more than 700 km to the east (Figure 1B). This isolated population represents either the only known, and possibly last, remnant of an entire lineage (*Desmognathus*) in the Ozark Plateau or an introduced species that could threaten local endemic species if it spreads (see Discussion).

There is a wealth of biogeographic evidence that suggests a relatively recent faunal connection between the Ozarks and the Appalachians, and many conspecifics or sister species of fish [30-32], crayfish [33] and salamanders [11,34,35] occur across these regions. On the other hand, surveys have shown *D. monticola *to be one of the most common salamander species found in fishing bait shops [36], so this population may have been introduced by fishermen or by other human activities. Without further detailed population genetic analyses, it is not possible to support or reject either scenario.

Using NCA, based on a portion of the mitochondrial gene cytochrome oxidase-1 (*cox1*), we test whether this isolate is the result of a recent human-mediated introduction or represents a relict from historical faunal connections between the Appalachian Mountains and the Ozark Plateau. If the Ozark population originated through fragmentation and contraction of a previously more widespread distribution, then we would expect it to exhibit significant genetic divergence from Appalachian populations. Alternatively, if the Ozark population results from a recent introduction through human activities, we would expect little or no genetic divergence from Appalachian populations, despite the large geographic separation between these highland regions. These predictions are ideally suited for testing with Nested Clade Analysis, which uses the geographic distributions of ancestral (interior) haplotypes relative to younger (tip) ones to draw inferences about processes that have shaped spatial patterns of genetic variation.

Adequately sampling genetic diversity is critical for testing phylogeographic hypotheses [37,38]. Although, given the fact that genetic diversity is not always randomly distributed across a species' distribution, it can be challenging to assess sampling adequacy *a priori*. Our sampling includes the entire latitudinal distribution of *D. monticola*, with dense sampling from the southernmost extent of the Appalachian Mountains where lineage diversity is known to be high for other co-distributed taxa [39-43]. We find that the highest genetic diversity in *D. monticola *is restricted to a localized region (< 2.5% of the distribution) in northern Georgia, and a correct diagnosis of the Ozark population is highly dependent on including critical haplotypes from this region in the NCA. We discuss our results in the context of developing sampling strategies based on comparative phylogeography of co-occurring species when assessing the history of populations that are suspected of being introduced.

## Results

**Figure 2:**
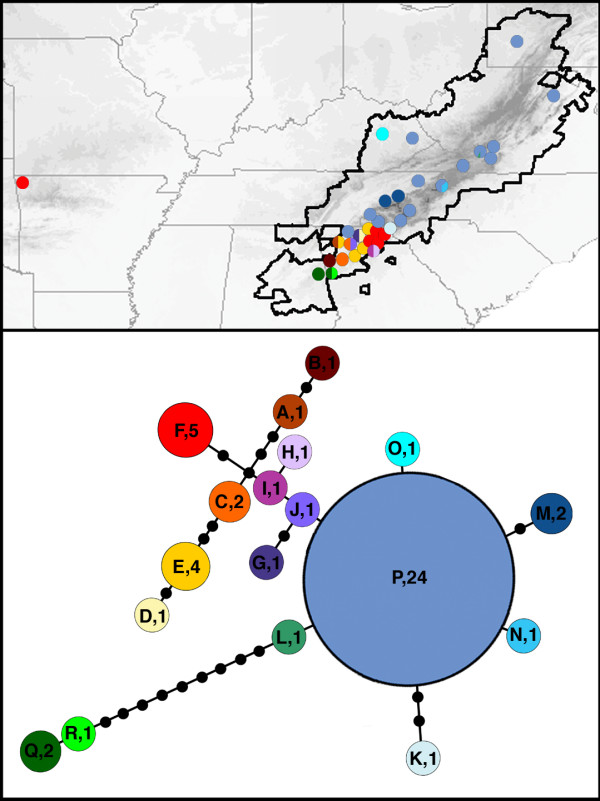
**Geographic distribution and unrooted statistical parsimony networks for *D. monticola *haplotypes**. The large majority of the geographic distribution contains only a few haplotypes (K, L, M, N, O, & P). The Ozark haplotype (F, red) occurs in the southern Appalachians of northern Georgia amongst a great diversity of related haplotypes. Labels on the network indicate the haplotype and the number of counties where it was found (sizes of circles are also drawn proportional to this number). Colors of haplotypes correspond to pie diagrams on the map and show the frequency of haplotyope in each county sampled. Black dots on haplotype network indicate hypothetical unsampled haplotypes.

**Figure 3:**
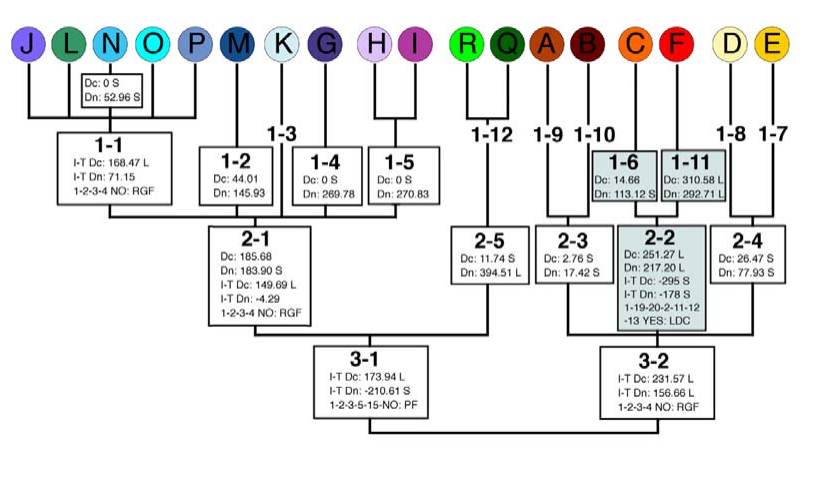
**Graphical summary of the nested haplotype structure and NCA**. Individual haplotypes are listed across the top, with increasingly more inclusive nested groups extending to the bottom. Interior haplotypes/nested groups are depicted in bold italics. Significant D_C_, D_N_, and I-T values are reported. Distances that are significantly small or large are indicated with S or L, respectively. The path taken through the most recent version of the inference key is shown; RGF, restricted gene flow; PF, past fragmentation; LDC, long-distance colonization; RE w/PF, range expansion coupled with past fragmentation. NCA results directly relevant to the diagnosis of the Ozark population (haplotype F) are highlighted in blue.

There was no nucleotide variation in the 515 basepair (bp) *cox1 *fragment among our seven Ozark samples. The 100 Appalachian samples from 47 populations covering the entire latitudinal distribution of *D. monticola *represented 18 unique *cox1 *haplotypes (Additional File 1). The geographic distribution of haplotypes was highly disproportional, with the highest diversity centered in the southern Appalachian Mountains (Figure 2, Table 1). Appalachian haplotypes ranged from 0.000% to 2.689% divergent from the Ozark population. Haplotypes identical (zero mutational steps; 0.000% divergent) to the Ozark population (haplotype F) were found in the region of highest haplotype diversity in northeast Georgia. The origin of the Ozark population of *D. monticola *was investigated with NCA [44,45] (See Methods Section). The distance among populations that have haplotype F is significantly large (Dc = 310.58, Dn = 292.71; Figure 3). The distance between haplotype F and its nearest neighbor in the network (haplotype C) is also significantly large (Dc = 251.27, Dn = 217.20). NCA of the clade including haplotypes C and F (clade 2-2) indicates that the Ozark population is the result of long distance colonization (i.e., Introduction). Our path to this conclusion is as follows. All nested clades are from separate areas (i.e., there is no geographic overlap between the distribution of haplotypes F and C; Yes to couplet #1). The species is present between where haplotypes F and C were found within the Appalachians (Yes to couplet #19), and we did sample this area (Yes to couplet #20). None of the conditions in couplets #2 or #11 are satisfied. There is a reversal in significance between I-T Dn and Dc values (I-T Dn = -178 S, Dc = 251.27 L; Yes to couplet #12). Since the Ozark population is separated from the geographical center of other clades (Yes to couplet #13), and there are no mutational differences between this population and some Appalachian populations, we conclude that the Ozark population arose from long distance colonization. Given the NCA evidence for restricted gene flow and dispersal within portions of the native range, and the limited dispersal capabilities of plethodontid salamanders [46,47], this long-distance movement likely resulted from human activities.

**Table 1:** Comparison of *cox1 *divergence between Appalachian and Ozark haplotypes. The Ozark population only contains a single haplotype (F). Note the large number of haplotypes restricted to northern Georgia (GA). See Figure two for map and Additional File 1 for locality details.

**Haplotype**	**uncorrected *p *to the Ozark population**	**Number of localities**	**States**
A	1.222%	1	GA
B	1.711%	1	GA
C	0.733%	2	GA
D	1.956%	1	GA
E	1.467%	4	GA
**F**	**0.000%**	**5**	**AR, GA**
G	0.978%	1	GA
H	0.978%	1	GA
I	0.733%	1	GA
J	0.978%	1	GA
K	1.956%	1	GA
L	1.467%	1	VA
M	1.711%	2	TN
N	1.467%	1	NC
O	1.467%	1	KY
P	1.222%	13	GA, KY, NC, PA, TN, VA, WV
Q	2.934%	2	AL, GA
R	2.689%	1	GA

State abbreviations are as follows:

AL = Alabama, AR = Arkansas, GA = Georgia, KY = Kentucky, NC = North Carolina, PA = Pennsylvania, TN = Tennessee, VA = Virginia, and WV = West Virginia.

**Figure 4:**
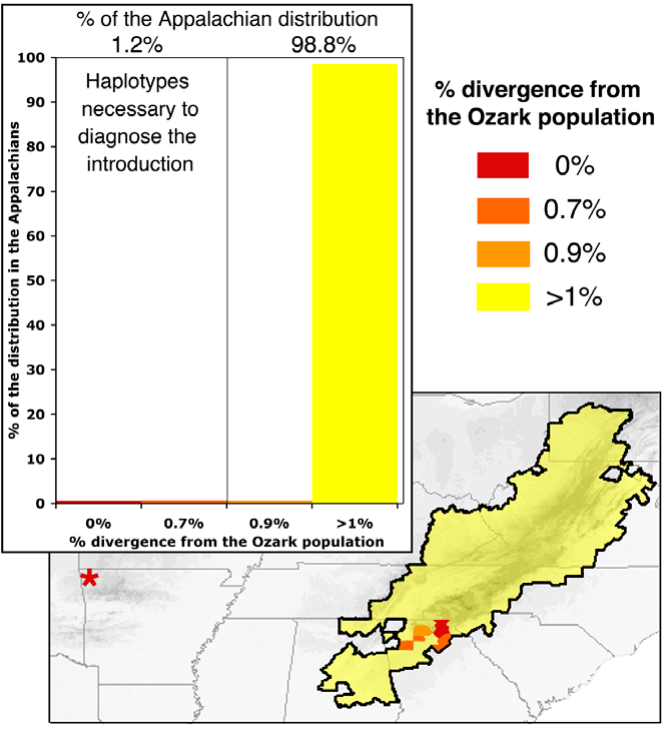
**Percent divergence of Appalachian haplotypes to the Ozark population and their geographic distribution**. Inset graph depicts the % sequence of the Appalachian haplotypes to the Ozark population and the % of the area of the distribution that they occur. The map shows the genetic landscape of haplotypes in relation to their divergence from the Ozark population. Note that it is necessary to include haplotypes that are ≤ 0.733% divergent from the Ozark population in order to diagnose it as introduced.

In order to test the necessity of including very similar (or identical) haplotypes to determine whether the Ozark population is native or introduced, we sequentially ran additional NCAs that excluded Appalachian samples that were 0.000% (haplotype F), and ≤ 0.733% (haplotypes F, C, and I) divergent from the Ozark population. Excluding only identical Appalachian haplotypes (Haplotype F = 0.56% of the distribution), still leads to the inference that the Ozark population was introduced. However, excluding Appalachian haplotypes F, C, and I, leads to a significantly small Dc for clade 2-2 (i.e., Ozark haplotype F). Since the Ozark population is separated from the remaining haplotypes by a larger than average number of mutational steps, and is allopatric from clades 2–3 and 2–4, this leads to the inference of allopatric fragmentation at the level of clade 3–2. This demonstrates that in this case it is necessary to locate and include in the NCA, haplotypes that are ≤ 0.733% divergent from the Ozark population, in order to properly diagnose the Ozark population as introduced. The distributions of these critical haplotypes (F, C, and I) are localized to a small region in northern Georgia that comprises < 1.2% of the geographic distribution of *D. monticola *(Figure 4).

## Discussion

### Distinguishing native populations from human introductions

To date most genetic studies of introduced species have focused on evolutionary genetics [48,49], genetic diversity [50], hybridization [51,52], and the identification of source populations of species that are known to be introduced [e.g., [50,53-57]. The field of phylogeography has played a major role in understanding patterns of species invasions [51,58]. However, there are relatively few studies that have tested whether or not a given species has been introduced by human activities [59-62], and little attention has been given to the approaches and pitfalls of molecular-based identification of putative invaders [63,64]. Here we demonstrate a case where the correct diagnosis of an isolated population (as native or introduced) using molecular methods is highly dependent on locating and sufficiently sampling a very restricted portion of the native distribution of a wide-ranging species. We identified this region and sampled it intensively based on the biogeographic history and patterns of genetic diversity of other co-distributed fauna.

Phylogeographic analyses based on inadequate sampling can lead to spurious results [37,38]. For many reasons, patterns of genetic variation are often non-randomly distributed across the species landscape. Therefore, the percentage of geographic distribution sampled does not necessarily equate to the percentage of existing haplotypes sampled, making it difficult to determine when adequate sampling has been achieved. Examining patterns of genetic diversity of co-distributed species can provide clues to patterns of diversity in the species of interest [65]. Many eastern North American species show considerable concordance in phylogeographic patterns, particularly due to the effects of Pleistocene glaciation [reviewed in [39]]. One phylogeographic pattern common to many animals, particularly salamanders, is that southern populations represent historical refugia and are genetically diverse while more northern populations underwent recent post-glacial range expansions and are often genetically uniform [e.gs., [20,39-43]. The phylogeographic patterns that we have elucidated for *D. monticola *are consistent with patterns exhibited by many co-distributed species. The large majority of the distribution of *D. monticola *represents a single lineage (containing the closely related haplotypes K, L, M, N, O, and P) that likely represents a recent range expansion from the southernmost highlands of the Appalachian Mountains all the way to the most northern extent of the distribution (Figure 2). The southern portion of the distribution may be a historical refuge and thus contains the highest genetic diversity. More than 50% of the *cox1 *haplotype diversity of *D. monticola *is restricted to less than 2.5% of the current geographic distribution of the species. In this case, the source of the human-mediated Ozark introduction is derived from this genetically diverse region in the southern Appalachian Mountains. When analyzing the isolated Ozark population of *D. monticola *in terms of a broad geographic sampling of its known native distribution, but only excluding Appalachian haplotypes that are ≤ 0.733%, the Ozark population appeared to be a unique historical remnant that deserves conservation attention (Figure 4). An analysis that contains a broad sampling from across the known native distribution, but only missing these critical haplotypes could look convincing, and subsequently lead to an incorrect diagnosis and the protection of an introduced species.

Comprehensive sampling of genetic diversity of the species' known native distribution may be essential for making an accurate assessment of the history of populations as native or introduced by humans, but exhaustive sampling may not be practical, especially when rapid identification is necessary for conservation strategies to be immediately implemented. We suggest that considering comparative phylogeographic patterns of co-distributed species can expedite an accurate diagnosis by acting as a guide to sampling the known native distribution of the species in question. For species that have undergone recent range expansions such as the one in this study, a comparative phylogeographic approach can help locate the areas of highest genetic diversity, and prevent over-sampling in genetically uniform regions. Furthermore, if phylogeographic data are available for co-distributed species with ecological requirements that are similar to those of the species in question (i.e., taxa potentially influenced by similar patterns of vicariance), this could effectively guide further sampling. For example, if the known native distribution of a terrestrial species extends across a region that is dissected by many rivers that are barriers to gene flow for other co-distributed terrestrial species, then initial analyses could compare the population in question to individuals from the land between each river (i.e., to each primary biogeographic subunit). This could help to narrow down which part of the distribution is genetically most similar to populations in question, and could help to focus subsequent sampling.

### Introduced Ozark Desmognathus

Based on our analyses it appears that the population of *D. monticola *recently discovered in the Ozark Plateau is introduced. We found that this population shares identical *cox1 *haplotypes with populations of *D. monticola *from four counties in the northeastern corner of the state of Georgia in the southern Appalachians (~1000 km away). Nested Clade Analysis of the *cox1 *fragment shows that this highly disjunct population results from a long distance dispersal (human-mediated introduction). Furthermore, we sequenced an additional 1.6 kb of a more variable region of mitochondrial DNA, upstream from our *cox1 *fragment, for select individuals. This region includes the gene NADH dehydrogenase subunit 2, tRNA^Trp^, tRNA^Ala^, tRNA^Asn^, tRNA^Cys^, tRNA^Tyr^, the origin for light-strand replication, and the beginning of *cox1*. We found only a single nucleotide substitution between the Ozark population and our samples from northeast Georgia (i.e., in total only one substitution in > 2 kb of mitochondrial DNA).

How did a southern Appalachian salamander get transported to the western Ozarks? In the southeastern United States, stream-dwelling plethodontid salamanders of the genera *Desmognathus*, *Gyrinophilus*, and *Pseudotriton *are vernacularly referred to as "spring lizards", and are commonly used as fishing bait for catching large species of centrarchid fishes such as largemouth bass (*Micropterus salmoides*). "Spring lizards" are collected from the wild and sold in bait shops by the thousands [66]. In fact, surveys of bait shops in northern Georgia (the source area of the Ozark introduction) found *D. monticola *to be the most common salamander sold [36]. Unfortunately, there is little information on the broader sales and distribution of "spring lizards" to investigate whether a bait bucket was the source of the Ozark introduction. This is not the only long-distance introduction of a salamander in the United States. North American tiger salamanders such as the barred tiger salamanders (*Ambystoma mavortium*) are also extensively collected and sold as fishing bait in the central and western United States. Populations of *A. mavortium *have been introduced to localities in California (~2000 km from their native distribution) where they hybridize with the rare California tiger salamander, *Ambystoma californiense *[52]. Another case is of the wandering salamander, *Aneides vagrans*, which was accidentally introduced to western Canada from central California through shipments of bark from oak trees in the early 1900s [60]. These salamanders have become well established and very widespread in coastal British Colombia and Vancouver, but are not noted to be invasive [60].

Do introduced *Desmognathus monticola *have a negative ecological impact in the Ozark Plateau? The Ozark Plateau is home to four stream/spring-dwelling species of plethodontid salamanders of the genus *Eurycea*:*E. lucifuga*, *E. longicauda melanopleura*, *E. spelaea*, and *E. tynerensis*, and the latter two (*E. spelaea *and *E. tynerensis*) are actually species groups that represent a major endemic Ozark radiation [24]. Although stream-dwelling *Desmognathus *and *Eurycea *are broadly sympatric in the Appalachian and Ouachita Mountains, the Ozark Plateau is geologically distinctly different from these regions [67] and contains very different stream habitats [68-70]. The Ozark Plateau is well drained and is very dry during the summer months. Moist streamside habitats that are important for some metamorphosing species of *Eurycea *as well as *D. monticola *are quite limited. *Desmognathus monticola *are large territorial salamanders and are very aggressive towards both conspecific and heterospecific stream-dwelling salamanders [71]. It has been hypothesized that in the southern Appalachians, where up to seven species of *Desmognathus *occur sympatrically [72], ecological boundaries between congeners are maintained by aggressive interference and predation [73-78]. *Desmognathus monticola *is larger and more robust than any of the Ozark *Eurycea*, and large adults would likely be the competitively-superior salamanders in a spring or stream. Although predation by large stream-dwelling *Desmognathus *on other salamanders in the wild is rare [79], this species has been shown to displace heterospecifics from the most suitable moist habitats [80]. Therefore, the presence of *D. monticola *could have a negative impact on Ozark *Eurycea *simply by excluding them from moist habitats at the periphery of springs and streams, a habitat feature that can be quite limited in arid areas of the Ozark Plateau.

Ozark *Desmognathus monticola *are currently known from only two locations. The salamanders included in this study are from a small spring ca. 1 meter wide that issues from the underside of a dirt bank and flows approximately 8.5 meters before reaching a much larger stream (Spavinaw Creek). Since the original discovery in 2003 we have visited this site four times and have found up to 25 individuals, ranging in size from very small newly metamorphosed juveniles, to very large adults (75+ mm SVL), in a single survey. Among the largest individuals, we found one gravid female, 55 mm SVL, with enlarged (ca. 2.5–3.0 mm in diameter) ovarian follicles (ca. 35 ova). Therefore, it appears that *D. monticola *are breeding and reproducing at this location. Recently, a second Ozark *D. monticola *location was discovered by members of the Arkansas Herpetological Society. Three adult *D. monticola *were found in a small spring entering Spavinaw Creek (36.4030°N, 94.3569°W) approximately 2.5 km upstream of the locality included in our analyses (K. Roberts *personal communication *2006). We presently know little about the abundance of *Desmognathus monticola *in the Ozarks and additional research on the ecological interactions between this introduced species and co-occurring species of *Eurycea *are necessary to determine the level of impact that it has on local salamander fauna.

## Conclusion

Our analyses of mitochondrial DNA indicate that the highly disjunct population of seal salamanders, *Desmognathus monticola*, recently discovered on the Ozark Plateau, is not native to the region. This population may need to be extirpated because of its potential negative impacts on endemic Ozark stream-dwelling salamanders. This conclusion could only be realized once nearly identical haplotypes from the known native distribution were included in our analyses. Locating the most genetically similar individuals (the putative source population) in the known native distribution of a species to effectively test the origin of an isolated population can be a labor-intensive and costly undertaking. We propose that considering comparative phylogeographic patterns can greatly facilitate this process and expedite diagnoses of native and introduced species.

## Methods

### Sampling

Salamanders from the Ozark population were collected from 30-September-2003 to 4-April-2005. Due to the limited distribution of the Ozark *Desmognathus*, vouchers were only taken for the first six individuals and were deposited at Arkansas State University Museum of Vertebrates (ASUMZ 28083–28086; 28156; 29032). Tail tips were taken in the field from subsequent specimens because at the time we did not know the species was introduced. These were compared to a large database of *D. monticola cox1 *sequences from GenBank [81,82], specimens collected from 8-May-2000 to 16-May-2002 by KHK that were deposited in the University of Minnesota's John Ford Bell Museum (JFBM), two tissue samples from the Museum of Vertebrate Zoology, University of California, Berkeley (MVZ), and samples collected in northern Georgia by JAW from 12-January-2006 and 26-March-2006 that were deposited in the MVZ (Additional File 1).

### DNA isolation, amplification and sequencing

DNA was extracted from fresh frozen tissues and ethanol preserved tissues using Qiagen DNeasy extraction kits. A portion of the mitochondrial gene cytochrome oxidase 1 (*cox1*, 515 bp not including primers) was amplified using the primers COX1F (5'-GGTATTGAGGTTTCGGTCTG-3') and COX1R (5'-CTTAGTCTCTTAATTCGAGC-3') [81] with standard protocols. PCR products were run out on 1.5 % agarose gels. Successful amplifications were cleaned with a Millipore PCR_96 _cleanup kit (Montáge™) or EXOSAPIT (USB Corp.). Big Dye (ABI) was used for cycle sequencing reactions sequenced on an ABI 3730 capillary sequencer. Sequencher ™ 3.1 (Gene Codes Corp.) was used to align and edit sequences. The alignment was unambiguous and the translation was checked in MacClade [83]. Sequences were deposited in GenBank; accession numbers are provided in Additional File 1. For the analyses we trimmed the fragment to 409 bp so that we could utilize sequences from GenBank. PAUP* 4.0b10 [84] was used to calculate uncorrected pairwise sequence divergences among populations. The sizes of the whole distribution of *D. monticola*, was based on the Global Amphibian Assessment [85]. We omitted a disjunct isolate from southern Alabama because preliminary mitochondrial DNA evidence suggests that it is a different species [81,82]. The size and percentage of the distribution of selected haplotypes was estimated based on the size of the county where they occur. This is actually a slight over estimate as multiple haplotypes occur in some counties, so in this species locating specific haplotypes to properly diagnose the introduction should be at least as difficult as we suggest if not more.

### Nested clade analysis

The origin of the Ozark population of *D. monticola *was investigated with Nested Clade Analysis [44,45] in order to test between two alternate hypotheses: past fragmentation (a natural historical relict) or long distance colonization (human-mediated introduction). Haplotype networks were constructed with statistical parsimony method implemented in TCS [86], and the haplotype networks were converted into nested clades [87,88]. The coordinates used in the analyses were either determined with a Garmin XL Global Positioning System (GPS) in the field, or extrapolated from topographical maps. The only locality information available for some of the GenBank sequences was the state and county, so we estimated the coordinates of the center point of the county for those samples. GeoDis version 2.0 [89] was used to calculate (1) the clade distance Dc, which measures the average distance of haplotypes in a nested group from its geographical center, (2) the nested clade distance Dn, which measures how far a haplotype group is from the geographic center of other groups with which it is nested, and 3) the average Dc and Dn separating interior and tip groupings of haplotypes. To test whether the geographic distributions of haplotypes were more widespread or restricted than expected by chance, we used a categorical permutation contingency analysis. The most likely historical and recurrent processes responsible for statistically significant patterns of phylogeographic variation were inferred using the revised inference key 11 November 2005 [45].

## Abbreviations

ASUMZ, Arkansas State University Museum of Zoology; bp, basepairs; *cox1*, cytochrome oxidase 1; Dc, clade distance; Dn, nested clade distance; I-T, interior to tip; JFBM, John Ford Bell Museum; MVZ, Museum of Vertebrate Zoology; NCA, nested clade analysis; PF, past fragmentation; RE, range expansion; RGF, restricted gene flow.

## Authors' contributions

RMB coordinated the study, collected and analyzed sequence data, and primarily prepared the manuscript. KHK contributed many Appalachian samples, performed the NCA, and contributed to the preparation of the manuscript. DRV assisted in collecting sequence data and contributed to the focus and preparation of the manuscript. AB discovered the Ozark population of *D. monticola *and participated in subsequent field trips. JAW collected invaluable material from across the state of Georgia and edited the manuscript. SET made the initial identification of AB's discovery and coordinated additional fieldwork and contributed to the preparation of the manuscript. All authors read and approved the final manuscript.

## Supplementary Material

Additional file 1 Locality information, museum and Genbank accession numbers for the *D. monticola *samples used in this study. Click here for file
