# Supplementary material for: The importance of comparative phylogeography in diagnosing introduced species: a lesson from the seal salamander, Desmognathus monticola
Source: BMC Ecol. 2007 Sep 7;7:7. doi: 10.1186/1472-6785-7-7 (PMC2020456; doi:10.1186/1472-6785-7-7)
Supplement: Additional file 1 — Locality information, museum and Genbank accession numbers for the D. monticola samples used in this study. [file 1472-6785-7-7-S1.doc]

**Additional File 1**

Specimen information

| Sample/Museum # or citation. | State | County | Latitude | Longitude | Haplotype | Genbank  Accession *cox1* |
| --- | --- | --- | --- | --- | --- | --- |
| Ozark population | | | | | | |
| ASUMZ 28083 | AR | Benton | 36.39900 | -94.38390 | F | EU034685 |
| ASUMZ 28084 | AR | Benton | 36.39900 | -94.38390 | F | EU034685 |
| ASUMZ 28085 | AR | Benton | 36.39900 | -94.38390 | F | EU034685 |
| Ozark tail tip #1 | AR | Benton | 36.39900 | -94.38390 | F | EU034685 |
| Ozark tail tip #2 | AR | Benton | 36.39900 | -94.38390 | F | EU034685 |
| Ozark tail tip #3 | AR | Benton | 36.39900 | -94.38390 | F | EU034685 |
| Ozark tail tip #4 | AR | Benton | 36.39900 | -94.38390 | F | EU034685 |
|  | | | | | | |
| Appalachian samples | | | | | | |
| [81] | AL | Clerburne | 33.71262 | -85.48756 | R | AF437460 |
| [82] | AL | Clerburne | 33.71262 | -85.48756 | R | AY549708 |
| MVZ 257269 | GA | Bartow | 34.27516 | -84.73164 | C | EU034682 |
| MVZ 257270 | GA | Bartow | 34.27516 | -84.73164 | C | EU034682 |
| MVZ 257284 | GA | Cherokee | 34.32599 | -84.32105 | E | EU034684 |
| MVZ 257285 | GA | Cherokee | 34.32599 | -84.32105 | E | EU034684 |
| MVZ 257271 | GA | Dawson | 34.38146 | -84.15205 | E | EU034684 |
| MVZ 257272 | GA | Dawson | 34.38146 | -84.15205 | E | EU034684 |
| MVZ 257295 | GA | Fannin | 34.75127 | -84.27531 | E | EU034684 |
| MVZ 257296 | GA | Fannin | 34.75127 | -84.27531 | E | EU034684 |
| MVZ 257282 | GA | Floyd | 34.57897 | -85.09805 | B | EU034681 |
| MVZ 257283 | GA | Floyd | 34.57897 | -85.09805 | B | EU034681 |
| JFBM 15190 | GA | Gilmer | 34.72280 | -84.37820 | D | EU034683 |
| JFBM 15191 | GA | Gilmer | 34.72280 | -84.37820 | D | EU034683 |
| JFBM 15193 | GA | Gilmer | 34.72280 | -84.37820 | D | EU034683 |
| MVZ 257297 | GA | Gilmer | 34.78047 | -84.32817 | G | EU034686 |
| MVZ 257298 | GA | Gilmer | 34.78047 | -84.32817 | G | EU034686 |
| MVZ 257290 | GA | Gordon | 34.61038 | -85.04812 | E | EU034684 |
| MVZ 257291 | GA | Gordon | 34.61038 | -85.04812 | A | EU034680 |
| MVZ 257292 | GA | Gordon | 34.61038 | -85.04812 | A | EU034680 |
| MVZ 257293 | GA | Hall | 34.36421 | -83.73825 | I | EU034688 |
| MVZ 257294 | GA | Hall | 34.36421 | -83.73825 | H | EU034687 |
| MVZ 257273 | GA | Lumpkin | 34.70827 | -83.91419 | F | EU034685 |
| MVZ 257274 | GA | Lumpkin | 34.70827 | -83.91419 | F | EU034685 |
| MVZ 257286 | GA | Murray | 34.48035 | -84.56589 | P | EU034695 |
| MVZ 257287 | GA | Murray | 34.48035 | -84.56589 | P | EU034695 |
| MVZ 257288 | GA | Pickens | 34.48815 | -84.48084 | J | EU034689 |
| MVZ 257289 | GA | Pickens | 34.48815 | -84.48084 | C | EU034682 |
| MVZ 257280 | GA | Polk | 33.93341 | -85.38120 | R | EU034697 |
| MVZ 257281 | GA | Polk | 33.93341 | -85.38120 | Q | EU034696 |
| [37] | GA | Rabun | 34.88912 | -83.43068 | K | AF437461 |
| MVZ 257276 | GA | Towns | 34.86370 | -83.76593 | F | EU034685 |
| MVZ 257277 | GA | Towns | 34.86370 | -83.76593 | F | EU034685 |
| MVZ 257278 | GA | Union | 34.68900 | -84.02210 | F | EU034685 |
| MVZ 257279 | GA | Union | 34.68900 | -84.02210 | F | EU034685 |
| MVZ 257275 | GA | White | 34.70936 | -83.78872 | F | EU034685 |
| [81] | KY | Floyd | 37.56488 | -82.77688 | P | AF437426 |
| JFBM 15212 | KY | Powell | 37.78250 | -83.67320 | O | EU034694 |
| JFBM 15200 | NC | Buncombe | 35.52520 | -82.51420 | P | EU034695 |
| JFBM 15201 | NC | Buncombe | 35.52520 | -82.51420 | P | EU034695 |
| JFBM 15202 | NC | Buncombe | 35.52520 | -82.51420 | P | EU034695 |
| JFBM 15208 | NC | Buncombe | 35.75480 | -82.47870 | P | EU034695 |
| JFBM 15209 | NC | Buncombe | 35.75480 | -82.47870 | P | EU034695 |
| No voucher | NC | Buncombe | 35.75480 | -82.47870 | P | EU034695 |
| JFBM 15189 | NC | Clay | 35.15080 | -83.76070 | P | EU034695 |
| JFBM 15182 | NC | Graham | 35.35830 | -83.71780 | P | EU034695 |
| JFBM 15183 | NC | Graham | 35.35830 | -83.71780 | P | EU034695 |
| JFBM 15184 | NC | Graham | 35.35830 | -83.71780 | P | EU034695 |
| JFBM 15185 | NC | Graham | 35.35350 | -83.90720 | P | EU034695 |
| JFBM 15186 | NC | Graham | 35.35350 | -83.90720 | P | EU034695 |
| JFBM 15187 | NC | Graham | 35.35350 | -83.90720 | P | EU034695 |
| JFBM 15188 | NC | Graham | 35.35350 | -83.90720 | P | EU034695 |
| JFBM 15195 | NC | Transylvania | 35.35330 | -82.77880 | P | EU034695 |
| JFBM 15196 | NC | Transylvania | 35.35330 | -82.77880 | P | EU034695 |
| JFBM 15197 | NC | Transylvania | 35.35330 | -82.77880 | P | EU034695 |
| JFBM 15198 | NC | Transylvania | 35.35330 | -82.77880 | P | EU034695 |
| JFBM 15199 | NC | Transylvania | 35.35330 | -82.77880 | P | EU034695 |
| MVZ 173495 | NC | Transylvania | 35.32170 | -82.79070 | P | EU034695 |
| [81] | NC | Watauga | 36.22480 | -81.70900 | P | AF437444 |
| [81] | NC | Watauga | 36.22480 | -81.70900 | N | AF437467 |
| No voucher | NC | Watauga | 36.21700 | -81.67500 | P | EU034695 |
| No voucher | NC | Watauga | 36.21700 | -81.67500 | P | EU034695 |
| [82] | NC | Watauga | 36.22480 | -81.70900 | N | AY549719 |
| MVZ 224966 | PA | Westmoreland | 40.11250 | -79.38280 | P | EU034695 |
| JFBM 15205 | TN | Cocke | 35.88300 | -83.19800 | M | EU034692 |
| JFBM 15206 | TN | Cocke | 35.88300 | -83.19800 | M | EU034692 |
| [81] | TN | Sevier | 36.20640 | -84.09256 | M | AF437451 |
| [81] | TN | Sevier | 36.20640 | -84.09256 | M | AF437452 |
| JFBM 15204 | TN | Unicoi | 36.17450 | -82.29880 | P | EU034695 |
| [81] | VA | Craig | 37.483182 | -80.21763 | P | AF437455 |
| [81] | VA | Craig | 37.483182 | -80.21763 | P | AF437437 |
| [82] | VA | Craig | 37.50583 | -80.17750 | P | AY549716 |
| [82] | VA | Craig | 37.50583 | -80.17750 | P | AY549690 |
| [82] | VA | Craig | 37.50583 | -80.17750 | P | AY549682 |
| [82] | VA | Craig | 37.50583 | -80.17750 | P | AY549689 |
| [82] | VA | Craig | 37.442609 | -80.33717 | P | AY549699 |
| [82] | VA | Craig | 37.442609 | -80.33717 | P | AY549684 |
| [82] | VA | Craig | 37.442609 | -80.33717 | P | AY549688 |
| [82] | VA | Craig | 37.494444 | -80.40697 | P | AY549695 |
| [81] | VA | Giles | 37.304649 | -80.71130 | P | AF437449 |
| [81] | VA | Giles | 37.304649 | -80.71130 | P | AF437448 |
| [82] | VA | Giles | 37.422780 | -80.55333 | P | AY549696 |
| [82] | VA | Giles | 37.422780 | -80.55333 | P | AY549694 |
| [82] | VA | Giles | 37.422780 | -80.55333 | P | AY549711 |
| [82] | VA | Giles | 37.304649 | -80.71130 | P | AY549698 |
| [82] | VA | Giles | 37.304649 | -80.71130 | P | AY549686 |
| [82] | VA | Giles | 37.198060 | -80.88667 | P | AY549718 |
| [82] | VA | Giles | 37.198060 | -80.88667 | L | AY549705 |
| [82] | VA | Giles | 37.441906 | -80.51946 | P | AY549691 |
| [82] | VA | Giles | 37.304649 | -80.71130 | P | AY549700 |
| [82] | VA | Giles | 37.304649 | -80.71130 | P | AY549712 |
| [82] | VA | Giles | 37.304649 | -80.71130 | P | AY549710 |
| [82] | VA | Giles | 37.304649 | -80.71130 | P | AY549692 |
| [82] | VA | Giles | 37.394332 | -80.54656 | P | AY549693 |
| [82] | VA | Giles | 37.304649 | -80.71130 | P | AY549683 |
| JFBM 15211 | VA | Grayson | 36.605700 | -81.51950 | P | EU034695 |
| [82] | VA | Montgomery | 37.35255 | -80.29202 | P | AY549703 |
| [82] | VA | Montgomery | 37.35255 | -80.29202 | P | AY549685 |
| [81] | VA | Montgomery | 37.35255 | -80.29202 | P | AF437435 |
| JFBM 15214 | VA | Rappahannock | 38.59950 | -78.26300 | P | EU034695 |

ASUMZ = Arkansas Museum of Vertebrate Zoology, Arkansas State University

JFBM = John Ford Bell Museum, University of Minnesota

MVZ = Museum of Vertebrate Zoology, University of California, Berkeley.

**References for Genbank samples:**

1. Rissler LJ, Taylor DR: **The phylogenetics of desmognathine salamander populations across the southern Appalachians.** *Molecular Phylogenetics and Evolution* 2003, **27:**197-211.
2. Rissler LJ, Wilbur HM, Taylor DH: **The influence of ecology and genetics on behavioral variation in salamander populations across the eastern continental divide.** *The American Naturalist* 2004,**164:**201-213.
